# Postharvest treatments with MnCl_2_ and ZnCl_2_ reduce enzymatic browning and enhance antioxidant accumulation in soya bean sprout

**DOI:** 10.1038/s41598-022-23367-7

**Published:** 2022-11-02

**Authors:** Xijun Jin, Weixin Zhou, Liang Cao, Yuxian Zhang

**Affiliations:** 1 College of Agriculture, Heilongjiang Bayi Agricultural University, 5 Xinfeng Road, Daqing, 163319 China; 2 National Coarse Cereals Engineering Technology Research Center, 5 Xinfeng Road, Daqing, 163319 China; 3 Key Laboratory of Soybean Mechanized Production, Ministry of Agriculture and Rural Affairs, 5 Xinfeng Road, Daqing, 163319 China

**Keywords:** Secondary metabolism, Nutrition

## Abstract

Soya bean sprout is a nutrient-abundant vegetable. However, enzymatic browning of soya bean sprouts during storage remains a challenge. In this study, the effects of treatment with MnCl_2_ or ZnCl_2_ on the browning index, antioxidant nutrient accumulation, total antioxidant capacity and enzyme activities of phenylalanine ammonia-lyase (PAL), polyphenol oxidase (PPO), peroxidase (POD), superoxide dismutase (SOD) and catalase (CAT) were investigated in soya bean sprouts after storage at 4 °C and 90% relative humidity for 0, 7, 14 and 21 days. The results showed that postharvest treatment with 1, 2 and 10 mM MnCl_2_ or ZnCl_2_ profoundly retarded enzymatic browning in soya bean sprouts to different extents. Compared with the control, the 10 mM MnCl_2_ and ZnCl_2_ treatments drastically enhanced ascorbic acid, total thiol and phenolic content, and enhanced FRAP (ferric-reducing ability of plasma) antioxidant capacity in stored soya bean sprouts. Moreover, the MnCl_2_ and ZnCl_2_ treatments enhanced SOD, CAT and PAL but decreased PPO and POD activities compared with the control. In addition, the Mn and Zn content in soya bean sprouts significantly increased, by approximately two- to threefold, compared with the control. This study provides a new method for improving the nutrient quality of soya bean sprouts based on postharvest Mn or Zn supplementation.

## Introduction

Germination is one of the most effective ways to improve the quality of legumes, which are important food sources in many countries^1,2^. Research has shown that germination and sprouting can produce secondary metabolites and antioxidant compounds (e.g., ascorbic acid, thiols and polyphenols) in vegetables^1,3^. Antioxidants are molecules that can scavenge reactive oxygen species and they play vital roles in preventing various human diseases and preserving food quality^4^. For example, dietary polyphenols contribute to the health benefits that plant-derived foods provide to humans^5^.

Soya bean (*Glycine max* L. Merr.) is used in various foods, such as soya milk and soya bean sprouts^6^. It contains a large amount of oil, proteins and bioactive compounds that can be lost during storage^2^. Enzymatic browning drastically reduces the sensory properties and marketability of vegetables and fruits during postharvest senescence^7–9^. Polyphenol oxidase (PPO, EC1.14.18.1) participates in the rapid degradation of polyphenols and produces brown byproducts, resulting in the postharvest browning of fruits and vegetables^10,11^. Other enzymes, such as phenylalanine ammonia-lyase (PAL, EC 4.3.1.1.) and peroxidase (POD, EC 1.11.1.7) are also involved in the enzymatic browning of postharvest fruits^12^. Soya bean contains abundant polyphenols and is highly susceptible to enzymatic browning, which can cause a drastic decline in polyphenol accumulation and a decrease in nutritional quality^13^. Many methods, including physical, chemical and natural antibrowning agents, have been developed to decrease the enzymatic browning of sprouts during storage^10,14,15^. For example, heat-shock treatment drastically decreases the enzymatic browning of mung bean sprouts^15^. However, no reports have shown the application of essential elements (e.g., Zn and Mn) in regulating the enzymatic browning of fruits and vegetables during storage.

Manganese (Mn) and zinc (Zn) are mainly obtained by humans from food and water and are vital for health^16,17^. For example, Mn and Zn are indispensable for human reproductive function^17^. These key mineral elements are also important for plant growth and development. Mn is required for many plant enzymes, such as malic, isocitrate dehydrogenase and PAL^18^. Zn is essential as a cofactor in many enzymes, such as superoxide dismutase (SOD, EC 1.15.1.1)^18^. Interestingly, previous research has shown that Mn and Zn can effectively inhibit PPO activities in plants^19^. Moreover, postharvest treatment with mineral elements can drastically delay jujube fruit senescence by enhancing antioxidant accumulation^20^.

In this study, soya bean sprouts were postharvest-treated with MnCl_2_ or ZnCl_2,_ and their quality was investigated during storage. We were confronted with two problems that needed to be addressed. First, could MnCl_2_ and ZnCl_2_ work as elicitors to decrease enzymatic browning in soya bean sprouts? Second, how do MnCl_2_ and ZnCl_2_ affect antioxidant accumulation in soya bean sprouts during storage? What are the possible mechanisms underlying this phenomenon? This study provides a new, simple method for improving soya bean sprout quality by inhibiting enzymatic browning and enhancing antioxidant accumulation during storage.

## Results

### Essential element accumulation

Postharvest treatment with 10 mM MnCl_2_ enhanced Mn accumulation by approximately 271% compared with the control (Table 1). Similarly, Zn accumulation increased by approximately 194% after treatment with 10 mM ZnCl_2_ in stored soya bean sprouts compared with the control (Table 1).

**Table 1:** Mineral element accumulation (mg kg^−1^ dry weight) in stored soya bean sprouts after spraying with 10 mM MnCl_2_ or ZnCl_2_. Means associated with the same letter are not significantly different for each element (*n* = 3; *P* < 0.05). *DW* dry weight.

	Water control	10 mM MnCl_2_	10 mM ZnCl_2_
Mn (mg kg^−1^ DW)	3.1 ± 0.3b	11.5 ± 0.7a	3.2 ± 0.2b
Zn (mg kg^−1^ DW)	4.7 ± 0.2b	4.6 ± 0.3b	13.8 ± 0.5a

### Enzymatic browning assay

**Figure 1:**
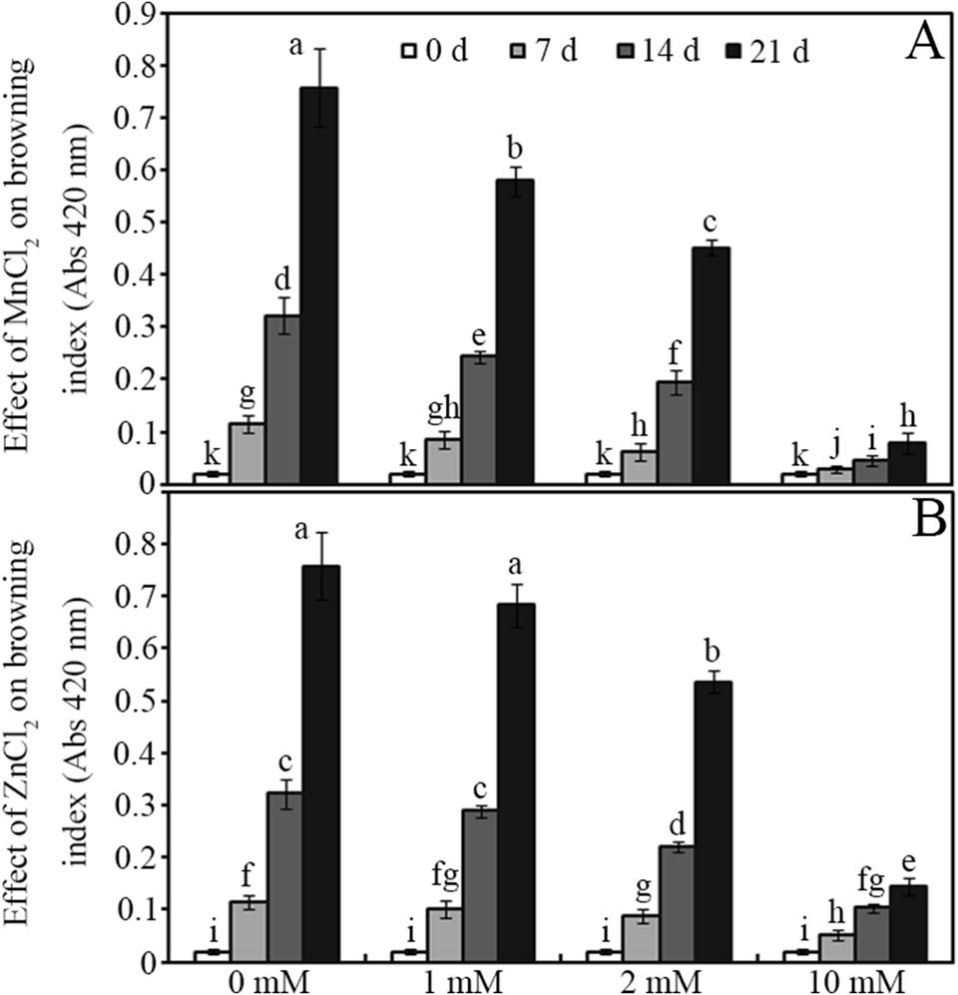
Effects of different concentrations (0, 1, 2 and 10 mM) of MnCl_2_ (**A**) and ZnCl_2_ (**B**) on enzymatic browning of soya bean sprouts after storage for 0, 7, 14 and 21 days. Bars represent the standard deviation of the mean (*n* = 3); means associated with the same letter are not significantly different (*P* < 0.05).

Both MnCl_2_ and ZnCl_2_ treatments significantly inhibited enzymatic browning in stored soya bean sprouts compared with that in the control (Fig. 1; *P* < 0.05). For example, the applications of 1, 2 and 10 mM MnCl_2_ decreased the browning index by approximately 24%, 40% and 90%, respectively, in soya bean sprouts compared with the control after storage for 21 days (Fig. 1A; *P* < 0.05). Compared with the control, 10 mM ZnCl_2_ decreased the browning index by approximately 55%, 68% and 81% in soya bean sprouts after storage for 7, 14 and 21 days, respectively (Fig. 1B; *P* < 0.05).

### Antioxidant nutrient accumulation and ferric-reducing ability of plasma (FRAP) antioxidant capacity

**Figure 2:**
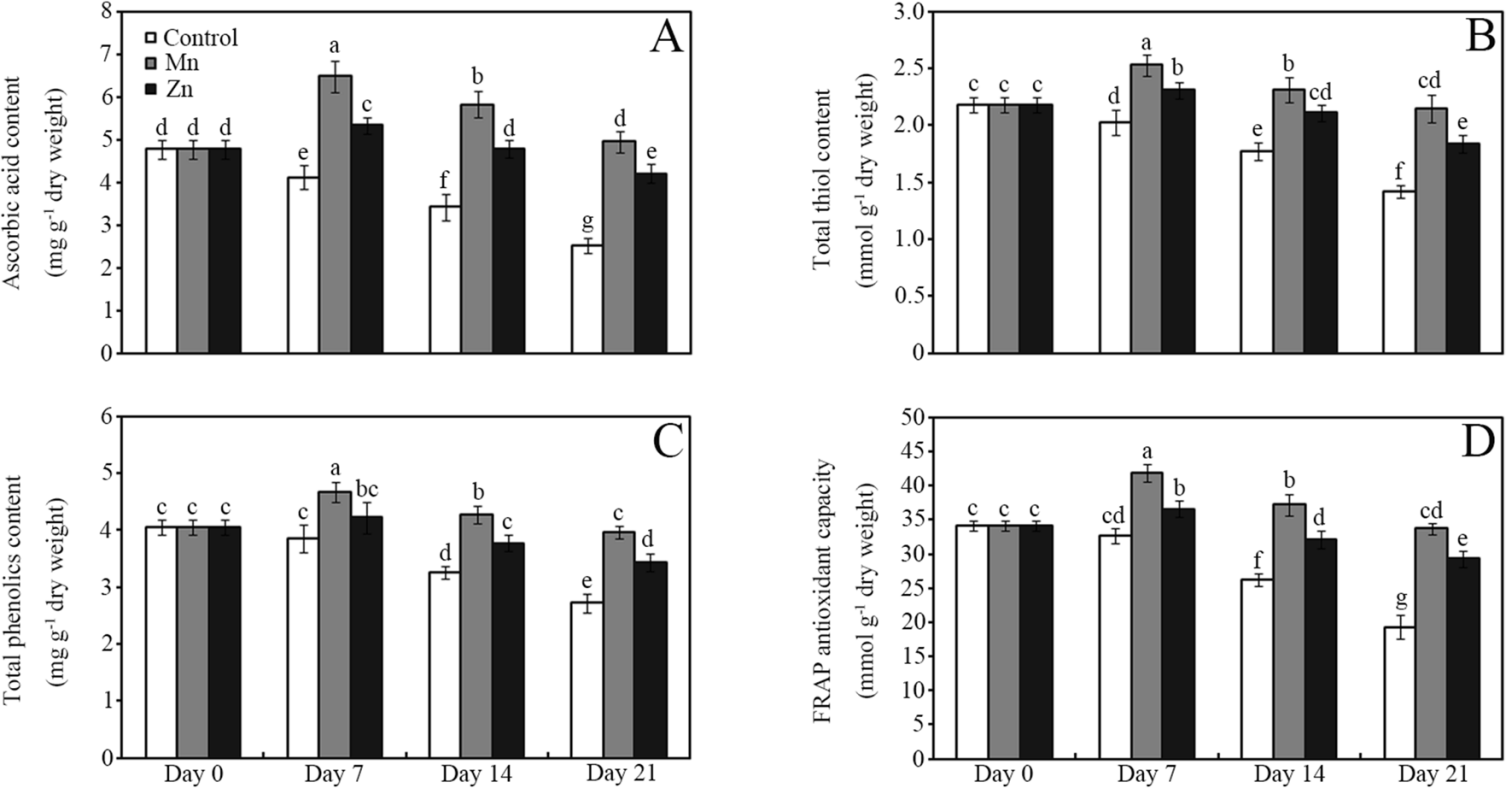
Effects of 10 mM MnCl_2_ and ZnCl_2_ on ascorbic acid level (**A**), thiol content (**B**), total phenolic content (**C**) and FRAP antioxidant capacity (**D**) of soya bean sprouts after storage for 0, 7, 14 and 21 days. Bars represent the standard deviation of the mean (*n* = 3); means associated with the same letter are not significantly different (*P* < 0.05).

Applying 10 mM MnCl_2_ or ZnCl_2_ significantly increased ascorbic acid, total thiol and total phenolic content and enhanced FRAP antioxidant capacity in the soya bean sprouts compared with the control (Fig. 2; *P* < 0.05). For example, treatment with 10 mM MnCl_2_ enhanced ascorbic acid content by approximately 57%, 70% and 96% compared with the control after storage for 7, 14 and 21 days, respectively (Fig. 2A; *P* < 0.05). Similarly, the application of 10 mM ZnCl_2_ enhanced total thiol content by approximately 14%, 19% and 30% in stored soya bean sprouts compared with the control after storage for 7, 14 and 21 days, respectively (Fig. 2B; *P* < 0.05). Compared with the control, treatment with 10 mM MnCl_2_ and ZnCl_2_ enhanced total phenolic content by approximately 46% and 26% in soya bean sprouts after storage for 21 days, respectively (Fig. 2C; *P* < 0.05). Similarly, the application of 10 mM MnCl_2_ and ZnCl_2_ enhanced FRAP antioxidant capacity by approximately 75% and 52%, respectively, in soya bean sprouts compared with the control after storage for 21 days (Fig. 2D; *P* < 0.05).

### Enzyme activities of soya bean sprouts

**Figure 3:**
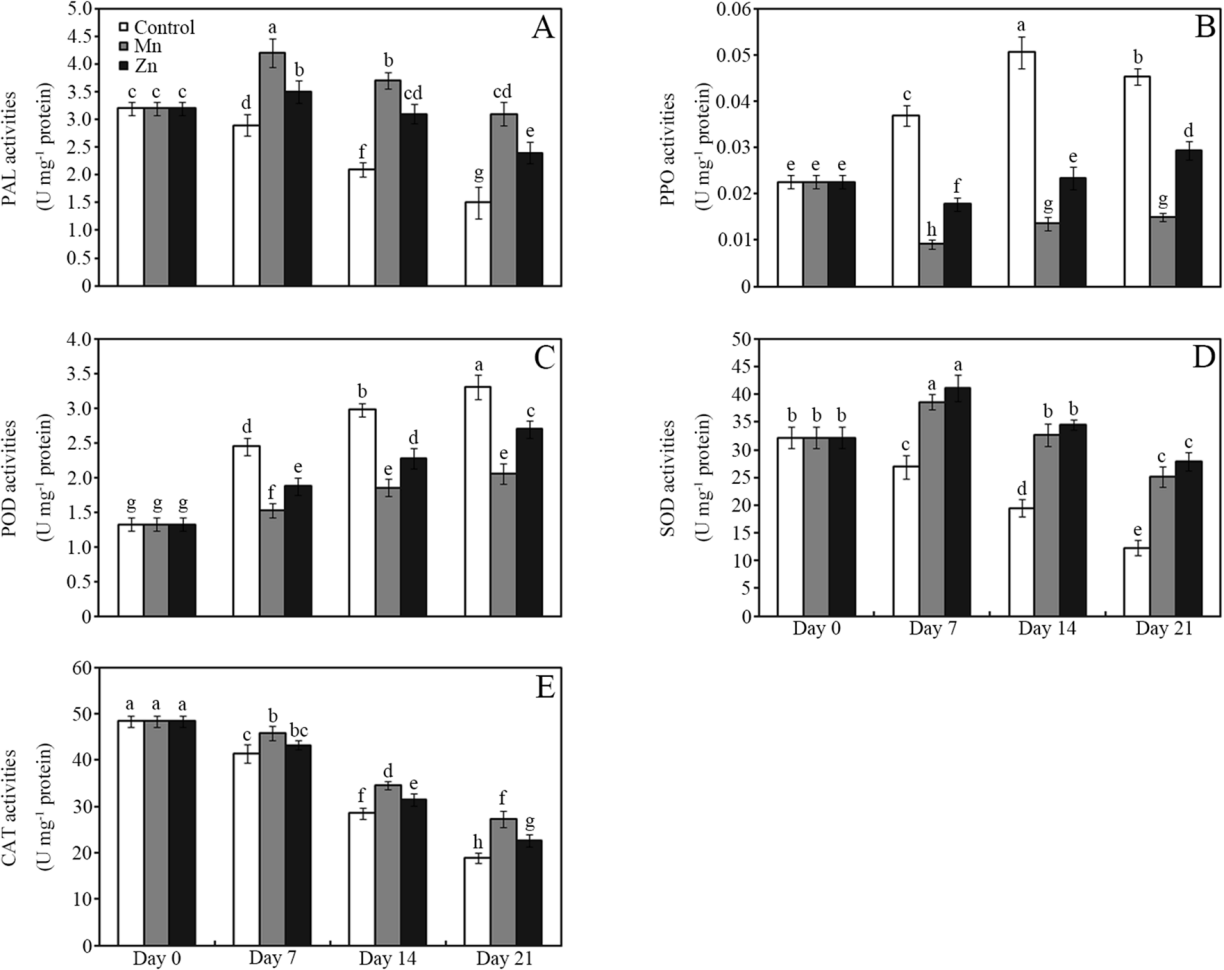
Effects of 10 mM MnCl_2_ and ZnCl_2_ on PAL (**A**), PPO (**B**), POD (**C**), SOD (**D**) and CAT activities (**E**) of soya bean sprouts after storage for 0, 7, 14 and 21 days. Bars represent the standard deviation of the mean (*n* = 3); means associated with the same letter are not significantly different (*P* < 0.05). *PAL* phenylalanine ammonia-lyase, *PPO* polyphenol oxidase, *POD* peroxidase, *SOD* superoxide dismutase, *CAT* catalase.

Applying 10 mM MnCl_2_ or ZnCl_2_ significantly affected PAL, PPO, POD, SOD and CAT activities in the soya bean sprout compared with the control (Fig. 3; *P* < 0.05). For example, treatment with 10 mM MnCl_2_ and ZnCl_2_ enhanced PAL activity by approximately 45% and 21%, respectively, in soya bean sprouts compared with the control after storage for 7 days (Fig. 3A; *P* < 0.05). Compared with the control, MnCl_2_ decreased PPO and POD activities by approximately 67% and 38%, respectively, in soya bean sprouts after storage for 21 days (Fig. 3B,C; *P* < 0.05). Similar changes were observed in the ZnCl_2_ treatment group. Moreover, the application of MnCl_2_ or ZnCl_2_ also enhanced SOD and CAT activities in soya bean sprouts compared with those in the control (Fig. 3D,E; *P* < 0.05). For example, MnCl_2_ enhanced SOD activity by approximately 43%, 68% and 104% in soya bean sprouts compared with the control after storage for 7, 14 and 21 days, respectively (Fig. 3D; *P* < 0.05). Similarly, MnCl_2_ and ZnCl_2_ increased CAT activity by approximately 44% and 20%, respectively, in soya bean sprouts compared with the control after storage for 21 days (Fig. 3E; *P* < 0.05).

## Discussion

Enzymatic browning is the second most common cause of quality loss in vegetables and fruits after that induced by microbiological contamination^21^. In this study, soya bean sprouts were postharvest-treated with 10 mM MnCl_2_ or ZnCl_2_ and enzymatic browning was drastically decreased after 3 weeks at 4 °C. This showed that both elements could be used for retarding enzymatic browning of stored soya bean sprouts. Studies have shown that abiotic stress can enhance ascorbic acid, thiol and phenolic biosynthesis and accumulation in plants^22–24^. For example, thiols play a central role in regulating heavy metal stress tolerance in plants^24^ and are indispensable for human health^25^. Thus, the effects of Mn and Zn on ascorbic acid, thiols, phenolic content and total antioxidant capacity were investigated in this study. The results showed that postharvest treatment with Mn could drastically increase ascorbic acid, total thiol and total phenolic content and enhance FRAP antioxidant capacity in soya bean sprouts compared with the control during storage for 21 days; the effect of Mn was greater than that of Zn. It is possible that Mn treatment induced greater oxidative stress than Zn treatment, eliciting greater antioxidant biosynthesis and accumulation in soya bean sprouts.

The results in this study suggest that Mn and Zn application can profoundly increase the accumulation of antioxidant nutrients, especially ascorbic acid, in stored soya bean sprouts. Interestingly, research has shown that postharvest treatment with ascorbic acid can profoundly enhance phenolic content but arrest enzymatic browning of stored mung bean sprouts^10^. Furthermore, the enhancement of nonenzymatic antioxidant accumulation could favor the inhibition of enzymatic browning in stored soya bean sprouts at low temperature. However, phenolics play an important role in the quality of plant-derived foods. Heavy metal stress can induce PAL, a key enzyme in phenolic biosynthesis^22^. In addition, PPOs and PODs are the main enzymes responsible for quality loss due to phenolic degradation^26^. In this study, postharvest treatment with Mn or Zn significantly inhibited enzymatic browning and enhanced phenolic accumulation in stored soya bean sprouts. Similarly, melatonin can delay postharvest fruit senescence by enhancing phenolic content and antioxidant enzyme activities (e.g., of SOD and CAT) but decreasing browning-related enzyme activities (e.g., of PPO and POD)^27^. This raised a question as to whether these enzymes are also involved in Mn-and Zn-regulated enzymatic browning in stored soya bean sprouts. Thus, the activities of phenolic-related enzymes such as PAL, PPO and POD were measured in the present study.

Higher PAL activity was observed in postharvest soya bean sprouts after treatment with 10 mM MnCl_2_ or ZnCl_2_ than in the control. PAL is a key enzyme in phenolic biosynthesis^28^. Thus, higher PAL activity promoted phenolic accumulation in soya bean sprouts compared with that in the control. This result is consistent with a previous report, which showed that heavy metals (e.g., Ni and Cu) can induce PAL activity and phenolic accumulation in plants^29,30^. However, lower PPO and POD activities were observed in the Mn- and Zn-treated soya bean sprouts than in the control. This result agrees with a previous report, which showed that Mn and Zn could significantly inhibit PPO activity in lentil (*Lens culinaris* Medik) sprouts^19^. In addition, Mn and Zn induced a higher antioxidant capacity, which could delay the senescence of the postharvest soya bean sprouts. One plausible explanation for the low POD activity could be the high antioxidant capacity that delays senescence^31^. Moreover, enhanced PAL activities, coupled with decreased PPO and POD activities, would also contribute to higher phenolic biosynthesis and accumulation in soya bean sprouts^12^. Thus, this Mn- and Zn-induced higher phenolic content, coupled with low PPO and POD activities, would lead to low enzymatic browning in stored fruits and vegetables^26^.

Moreover, the activities of antioxidant enzymes such as SOD and CAT were also measured. Compared with the control, SOD and CAT activities drastically increased after treatment of stored soya bean sprouts with MnCl_2_ or ZnCl_2_. As for the possible mechanism, one plausible explanation is that Zn and Mn are key components of SOD and CAT enzymes^32^. Activation of antioxidant enzymes (e.g., SOD and CAT), as well as the accumulation of nonenzymatic antioxidants (e.g., ascorbic acid and phenolics), could delay senescence and improve postharvest soya bean sprout quality. This is consistent with previous reports, which showed that H_2_S maintains good nutritional value and delays postharvest senescence in postharvest tomato fruits by enhancing antioxidant enzyme activity^33^.

In addition, a high accumulation (a two- to threefold increase) of Mn and Zn was observed in MnCl_2_- and ZnCl_2_-treated soya bean sprouts compared with the control. This agrees with a previous report, which showed that soya bean seeds soaked with Mn and Zn could produce mineral element-rich sprouts^34^, suggesting that postharvest treatment, as well as seed soaking, could efficiently enhance mineral element accumulation in soya bean sprouts. Moreover, this enhanced elemental content, coupled with antioxidants, would further improve the nutritive value of soya bean sprouts to consumers.

In conclusion, postharvest treatment of stored soya bean sprouts with MnCl_2_ or ZnCl_2_ could profoundly delay enzymatic browning by inhibiting the activities of phenolic-related enzymes such as PPO and POD, coupled with the accumulation of nonenzymatic antioxidants such as ascorbic acid, thiols and phenolics, and the increase of antioxidant enzyme activities (e.g., of SOD and CAT). In addition, postharvest treatment with MnCl_2_ or ZnCl_2_ could also enhance Mn and Zn content in soya bean sprouts compared with the control. This study provides a new method for improving soya bean sprout quality through postharvest treatment.

## Methods

All local, national or international guidelines and legislation were followed during this study.

### Experimental design

Soya bean seeds were provided by Daqing Sazhong Seed Co. (Daqing, China). Seeds of identical size and weight were selected, sterilized with 1% NaClO for 10 min, rinsed and then steeped in distilled water at room temperature for 2 h. Subsequently, they were placed on trays filled with fine sand (washed with distilled water daily) before being kept in a dark chamber at 22 ± 2 °C and 70% relative humidity. Sprouts of the same length (9.2 ± 0.6 cm) and weight (472 ± 35 mg/sprout) were selected and harvested on day 7 after sowing. Before storage, soya bean sprouts without disease (infection) were selected, washed with sterilized water and then soaked with 1, 2 and 10 mM MnCl_2_ or ZnCl_2_ (distilled water as a control) for 30 min, respectively. Subsequently, the treated soya bean sprouts were washed with distilled water and kept in polypropylene boxes for 0, 7, 14 and 21 days at 4 °C and approximately 90% relative humidity. Soya bean sprouts without disease symptoms were frozen in liquid N_2_ immediately and kept at − 70 °C for subsequent analysis of mineral element content, ascorbic acid level, total thiol content, total phenolic accumulation, FRAP antioxidant capacity, and enzyme (PAL, PPO, POD, SOD and CAT) activities. Three replicates of 100 seeds were included for each treatment for all assays. All concentrations used in this work were based on our preliminary research.

### Essential element assay

Mineral content (Mn and Zn) was determined by the method reported by Fiket et al.^35^ using high-resolution inductively coupled plasma mass spectrometry with a Thermo Fisher Scientific HRICP-MS Element 2 instrument equipped with an ESI-a SC-2 DX FAST autosampler and indium as an internal standard. Before analysis, powdered lyophilized samples were subjected to microwave-assisted acidic digestion in HNO_3_/HF (60:1, v/v) using a Multiwave 3000 at 1400 W.

### Browning index assay

The browning index of soya bean sprouts was measured using the method of Kim et al.^36^. Briefly, 10 g of fresh soya bean sprouts were homogenized in 100 mL trichloroacetic acid (10%), allowed to stand for 2 h at 35 °C, and centrifuged at 15,000×*g* at 25 °C for 15 min. The supernatant was collected, and the absorbance was recorded at 420 nm.

### Antioxidant assay

A titrimetric method with 2,6-dichlorophenol-indophenol (2,6-DPI) was used to assess the ascorbic acid (vitamin C) accumulation level in soya bean sprouts^37^. Ascorbic acid solution (0.05%) was used to calibrate the 2,6-DPI solution. The results are expressed as mg of ascorbic acid equivalents per g of dry weight. All assays were completed within 10–15 min.

The total thiol content of soya bean sprouts was analyzed using the method of Nagalakshmi and Prasad^38^. Soya bean sprouts were homogenized in 20 mM EDTA in an ice bath and filtered. Briefly, 0.5 mL of homogenate was mixed with 1.5 mL of 100 mM Tris buffer (pH 8.2) and 0.1 mL of 10 mM 5,5′-dithiobis (2-nitrobenzoic acid) (DTNB). Then, the mixture was diluted to 10 mL by adding 7.9 mL of absolute methanol. After development for 15 min, the absorbance of the clear supernatant was measured at 412 nm. Total sulfhydryl groups were calculated using the extinction coefficient of 13,100.

The total phenolic content of the soya bean sprouts was determined using the method described by Singleton and Rossi^39^. The Folin–Ciocalteu reagent method was used to measure the total phenolic content and the absorbance was recorded at 760 nm. The results are expressed as gallic acid equivalents (mg g^−1^ of dry weight).

### FRAP antioxidant capacity assay

Soya bean sprouts (5 g) were immediately frozen in liquid N_2_ and ground into a powder, which was transferred to 0.1 L ethanol–water solution (50%, v/v) and incubated in the dark (4 °C for 48 h). The filtrates of the extracts from each replicate were mixed, and the solvent was removed. The extracts were stored at 4 °C for total antioxidant capacity analysis using the FRAP assay^40^. FeSO_4_ solution was used to construct a standard curve, and the results are expressed in mM Fe(II) g^−1^ dry weight.

### Defense enzyme assay

SOD activities in soya bean sprouts were measured using the method of Dhindsa et al.^41^. Different volumes (0, 50, 100, 150 and 200 µL) of enzyme extract, combined with 0.1 M phosphate buffer (pH 7.8), were added to the reaction buffer. After exposure of the mixture to light for 15 min, the increase in absorbance at 560 nm due to formazan formation was recorded. The amount of enzyme that inhibited 50% of the nitro blue tetrazolium photoreduction was defined as one unit (U) of SOD activity.

Catalase (EC 1.11.1.6) activity was determined using the method of Zhang and Kirkham^42^. Reaction buffer (3 mL) was mixed with 50 μL enzyme extract from the soya bean sprouts, and then the reaction was started by adding 15 mM H_2_O_2_. The activity was measured by monitoring the consumption of H_2_O_2_ at 240 nm (E = 39.4 mM^−1^ cm^−1^) for 3 min. The amount of enzyme catalyzing the decomposition of 1 µmol of H_2_O_2_ per min per mg of protein was defined as one unit of CAT activity.

The activity of POD in soya bean sprouts was measured using the guaiacol method of Khalil et al.^43^. The reaction mixture was composed of 10 mM phosphate buffer (pH 7.0, 10 mM H_2_O_2_, 20 mM guaiacol) and 0.5 mL crude extract in 3 mL. The increase in absorbance due to the dehydrogenation of guaiacol was measured at 470 nm using a Spectronic 601 UV–vis spectrophotometer. The amount of enzyme catalyzing the formation of 1 µmol of tetraguaiacol per min per mg of protein was defined as one unit of POD activity.

The activity of PAL in soya bean sprouts was measured at 290 nm using the method of Dickerson et al.^44^. Soya bean sprouts (1 g) were homogenized with 10 mL of ice-cold sodium borate buffer (0.1 M, pH 8.7) using a mortar and pestle. The homogenate was centrifuged at 10,000×*g* for 30 min at 4 °C, and the supernatant was collected for the enzyme activity assay, which was based on the rate of conversion of l-phenylalanine to *trans*-cinnamic acid. The amount of enzyme catalyzing the formation of 1 µmol of cinnamic acid per min per mg of protein at 25 °C was defined as one unit of PAL activity.

The activity of PPO was measured using the method of Gawlik-Dziki et al.^8^ Samples of soya bean sprouts (2 g) were ground with 8 mL sodium phosphate buffer (0.1 M, pH 5.8) containing 0.2 g polyvinyl-polypyrrolidone. Extracts were homogenized and centrifuged at 12,000×*g* at 4 °C for 30 min, and supernatants were collected. Next, 0.1 mL of supernatant was incubated with 2 mL of sodium phosphate buffer (pH 5.8) and 0.5 mL of catechol (0.5 M) at 25 °C for 5 min, and the absorbance was measured at 420 nm. One unit of PPO activity was defined as a change of 0.001 in absorbance per min.

Soluble protein concentration was measured by the method of Bradford^45^ using bovine serum albumin as a standard.

### Data analysis

All experiments were conducted in a completely randomized design, with three replicates per treatment. All data were analyzed using Duncan’s multiple range test using SPSS software v13.0 (IBM Corp., Armonk, NY, USA) at *P* ˂ 0.05.

## Data Availability

All data analyzed during this study are included in this published article. The detailed data are available on reasonable request from the corresponding author.
